# Towards intelligent railway monitoring: A novel hybrid deep learning architecture for railway obstacle detection

**DOI:** 10.1371/journal.pone.0349562

**Published:** 2026-05-29

**Authors:** Christopher Mai, Luca Eisentraut, Merlin Schadt, Ricardo Buettner

**Affiliations:** Chair of Hybrid Intelligence, Helmut-Schmidt-University/University of the Federal Armed Forces Hamburg, Hamburg, Germany; Leibniz University Hannover, GERMANY

## Abstract

Railways are among the most efficient modes of transportation, capable of moving large quantities of goods and passengers over long distances at relatively low cost. However, accidents frequently occur due to objects or individuals present on the tracks, as trains are unable to swerve and require long braking distances. While the localization of objects within the track bed is a well-explored topic, the reliable and high-performance classification of such obstacles across all relevant categories remains an unresolved challenge. This study proposes an innovative hybrid architecture that leverages the specific visual characteristics of track bed imagery, setting a new benchmark in this domain. The hybrid design effectively leverages the strengths of ResNet50 and Swin Transformer V2, allowing the model to capture both local and global features. In addition, an Efficient Attention Module is integrated to further emphasize the most relevant features for robust obstacle classification. Using stratified five-fold cross-validation on a dataset of 2,003 images across six classes (iron bar, boulder, person, branch, canister, and barrel), the model achieved an average balanced accuracy of 99.46%. The results have implications for accident prevention, improving operational efficiency, and modernizing railway safety systems, thereby enabling the future application of automatic railway surveillance systems to ultimately enhance operational security.

## 1 Introduction

Railroads are acknowledged as one of the most efficient modes of transportation due to their capacity to transport substantial quantities of goods and individuals over extended distances at a high rate of speed at a relatively low cost [1]. This efficiency is crucial for sustainable development and reducing environmental impacts [1]. Due to this high usage rate of railway systems, significant incidents involving people or objects on railroad segments occur every year [2], including 1,507 significant railway accidents in 2024, resulting in the deaths of 750 individuals and the severe injuries of an additional 548 individuals [3]. Therefore, there is a need to monitor all relevant parts of railway infrastructure, including the track bed. The presence of objects in this area can result in a range of adverse outcomes, including derailments, collisions, service delays, and in extreme cases, the loss of human life [3]. Traditionally, such monitoring relied heavily on manual inspections by workers and security personnel [4]. Even in highly developed regions where cameras have been implemented, these systems often fail to provide comprehensive coverage and timely detection and classification of hazards, resulting in persistent safety risks and financial burdens for railway operators [4]. Recently, the use of deep learning techniques has significantly enhanced innovation in this domain. Some works employ lightweight deep learning models, such as those by Chen et al. [5] and Ye et al. [6], which achieve high accuracy for real-time track segmentation and obstacle detection without heavy computational demands. UAVs and multisensor systems, like RSNet [7] and YOLO-UAT, leverage aerial and LiDAR [8] data to improve obstacle and foreign object detection, even in challenging conditions such as low light or complex imagery. Additionally, high-computing approaches utilize YOLO architectures with attention mechanisms and efficient layers, boosting small-object detection accuracy and enabling real-time deployment on embedded systems [9,10]. Finally, real-time and video-based methods, like RailNet [11] and modified YOLOv3 [6], provide fast, accurate track monitoring and object localization, supporting the modernization of railway safety systems.

**Fig 1:**
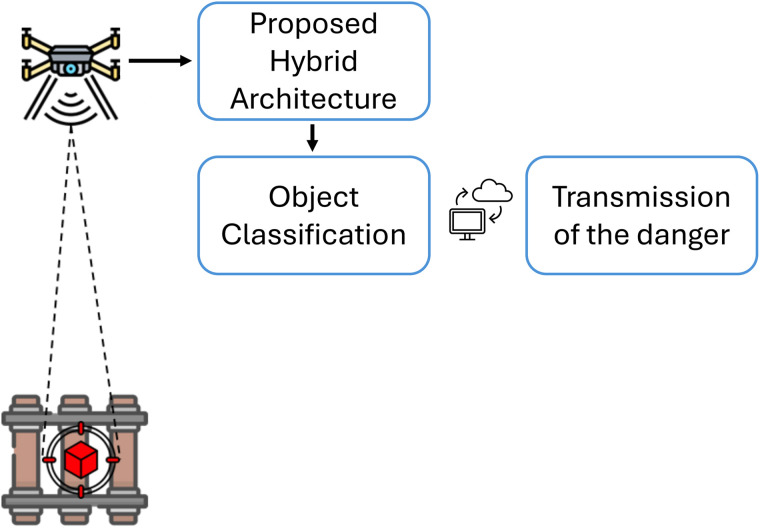
Example of a potential application of our hybrid deep learning approach, in which a drone identifies objects on railway tracks to enhance safety and prevent accidents.

However, to the best of our knowledge, no existing approach is able to classify robustly all relevant classes of objects in the track bed, therefore leaving a research gap. This gap is crucial since it is more relevant to classify objects in the track bed to determine whether emergency braking is needed, rather than knowing the precise location of potential foreign objects. To address this gap while achieving very high performance, an approach is needed that is tailored to the specific problem domain. Objects in the track bed are characterized on the one hand by their global shape and on the other hand by local characteristics such as textures. In order to leverage these characteristics, this study proposes an innovative hybrid model consisting of ResNet50 and a Swin TransformerV2 backbone enhanced with an efficient channel attention (ECA) module (Fig 1). To test whether the model is able to robustly classify all relevant classes of obstacles, the most diverse dataset from Rampriya et al. [12] (six classes, 2,003 images) and a stratified 5-fold cross-validation are used. This yields an average balanced accuracy of 99.46% and thus sets a new benchmark, resulting in three contributions:

1. We propose an innovative hybrid architecture that achieves a new benchmark of 99.46% for the classification of obstacles in the track bed.
2. We show that hybrid models deliver particularly high performance for classification tasks by addressing different aspects of the problem.
3. We emphasize the importance of the interaction of convolutional neural networks and visual transformers to achieve the highest classification accuracies.

The rest of the paper is structured as follows: First, we highlight related work before the methodology provides a detailed description of the proposed hybrid deep learning architecture. The results section presents a comprehensive evaluation of the model’s performance before discussing and contextualizing these results.

## 2 Related work

### 2.1 Monitoring railways

In addition to threats to operational safety due to gradually developing damage such as wear and tear, abruptly occurring obstacles pose a more immediate threat. While the former can be controlled through regular maintenance, there is no ideal solution for mitigating the latter. In general, one faces a dilemma: if the aim is to monitor the entire route network, a scalable approach must be chosen. This can consist of passive, low-cost measures such as the installation of protective fences [13], or in the operating instruction to run trains at reduced speed in order to detect potential obstacles directly from the driver’s cab. The other option is to monitor only critical points such as level crossings, which ensures a high level of operational safety there, but leaves other parts completely unmonitored. In practice, a middle way is chosen: particularly critical points such as platforms and level crossings are monitored especially closely. However, as foreign objects on the railway can lead to delays due to the removal of these objects or to collisions and thus possibly to derailments and personal injury [3,5], sections of track are also monitored in some cases. This is currently done primarily by monitoring train dispatchers to avoid collisions with other trains [14], but also by using cameras along the route. Data collected in this way is currently often evaluated to a large extent by humans [15], which significantly impairs scalability.

A practical, automated and scalable system for monitoring railways must therefore be established. The primary requirement for this is performance. If this is not reached at a near-perfect level, an application is not economical or safe due to false alarms or missed classifications. The second priority is the classification of the object, as non-optical methods such as induction loops or similar techniques are not yet in widespread use, the decisive criterion is the visual appearance of the obstacle. Based on this, a decision must be made as to whether an intervention is necessary or whether it is merely a visually extensive but harmless obstacle (e.g., leaf cover over the rails). The exact position of the obstacle on the rails is less important, as this would only be required if the obstacle were to be removed automatically. As a result, a high-performance classification approach for obstacles on railway tracks based on optical images is urgently needed.

### 2.2 Deep learning in railway monitoring

The need for a high-performance classification approach for obstacles on railway tracks based on optical images described above can be addressed very well by such CNNs. In addition to a binary **classification**, such systems can also perform a classification into three or more classes or detect the location of objects by using bounding boxes (**object detection**). Finally, these networks can also be used in the present application by **segmenting** targeting objects on the input image with pixel accuracy.

#### 2.2.1 Segmentation.

Segmentation is considered the most complex of the three categories mentioned and can be semantic (e.g., all persons in the track bed as one label) or instance-based (e.g., three persons are labeled as three different ones). Various works in the relevant literature address the segmentation problem: Chen et al. [5] use a You-Only-Look-Once (YOLO) based model to segment the tracks from the rest of the background and then detect potential interfering objects. This widely used approach simultaneously recognizes which type of object is located where in the image in a single pass. The architecture developed by the authors on this basis and extended by a transformer achieves a mean Intersection over Union (mIoU) of 92.5% on the so-called RailSem19 dataset, which is often used for training and validation of models in this problem domain. Also, Gao et al. [16] segment disturbance objects such as people in the track bed. The authors use a dataset with approx. 5,000 samples and can achieve a mean average precision (mAP) of 98.85%. Wang et al. [17] take a similar approach to [5] and segment the rail itself from the image background based on 3,000 images from actual operation. The authors can achieve a slightly lower mIoU of 89.8%. Finally, images taken by a drone can also be used. Rampriya et al. [7] use 2,214 samples of such a dataset to segment the trace from the image background and can achieve 0.973 as dice coefficient based on a three-class problem.

#### 2.2.2 Object detection.

The automatic placement of one or more bounding boxes on the image to be evaluated and thus the so-called object detection is used by the largest number of relevant publications [6,9,18–25]. Using the already mentioned RailSem19 dataset, Sevi et al. [22] achieve an mAP@50, i.e., a mean average precision at IoU threshold 0.50, of 88.8%. The authors recognize people or cars in the samples. A broader set of classes, seven, is addressed by Ye et al. [6] using 8,776 images. Based on a fixed train-test-split, an mAP of 94.75% can be achieved, whereby the lightweight model used by the authors recognizes pedestrians, for example. The most diverse dataset of the relevant studies is used by Zhang et al. [19], whose samples contain 12 groups and comprise over 18,000 images. The authors achieve an mAP@0.5 of 77.5% and recognize, for example, buses, bicycles, people, and motorcycles. Another, more practice-oriented approach is taken by He et al. [18], who do not use a widely established dataset but capture images in Chinese subways to detect objects at a sufficient distance for emergency braking. A performance of 93.00% can be achieved.

#### 2.2.3 Classification.

Finally, a few papers focus on the task of this study, the classification of images. Chen et al. [26], for example, use 3,145 images labeled as “intruded” to perform a binary classification. This intruded class could be distinguished from the baseline (non-intruded) class with a mean precision of 96.88%, which shows that, e.g., bottles or umbrellas are basically recognized as interference by the algorithms. Fayyaz and Johnson [27] tackle a similar task, but only for level crossings. They use a transfer-learning approach based on ImageNet to classify bicycles or pedestrians at such locations, for example. The algorithm, fine-tuned to the specific problem, achieves an accuracy of 88.00%. No real classification, object recognition, or segmentation is applied in the work by Wang et al. [11], who instead perform anomaly detection, achieving an Area Under the Receiver Operating Characteristic Curve (AUC-ROC) of 85.66%. Trained on 6,656 images of inconspicuous track beds and tested on 316 abnormal images (579 images in total), the authors show good model performance. Kapoor et al. [28] is the only publication in the literature core that does not use optical images, but 749 thermal images. The authors achieve an accuracy of 85.2% in the binary classification of images. In contrast to object recognition and segmentation, the classification of images is therefore a less addressed field for the problem at hand. However, as shown above, from a practical point of view, it is more relevant to achieve higher classification accuracy than lower performance in object recognition. This results in the research gap, which is the starting point of the present work.

## 3 Methodology

### 3.1 Model architecture

#### 3.1.1 Hybrid architecture.

**Fig 2:**
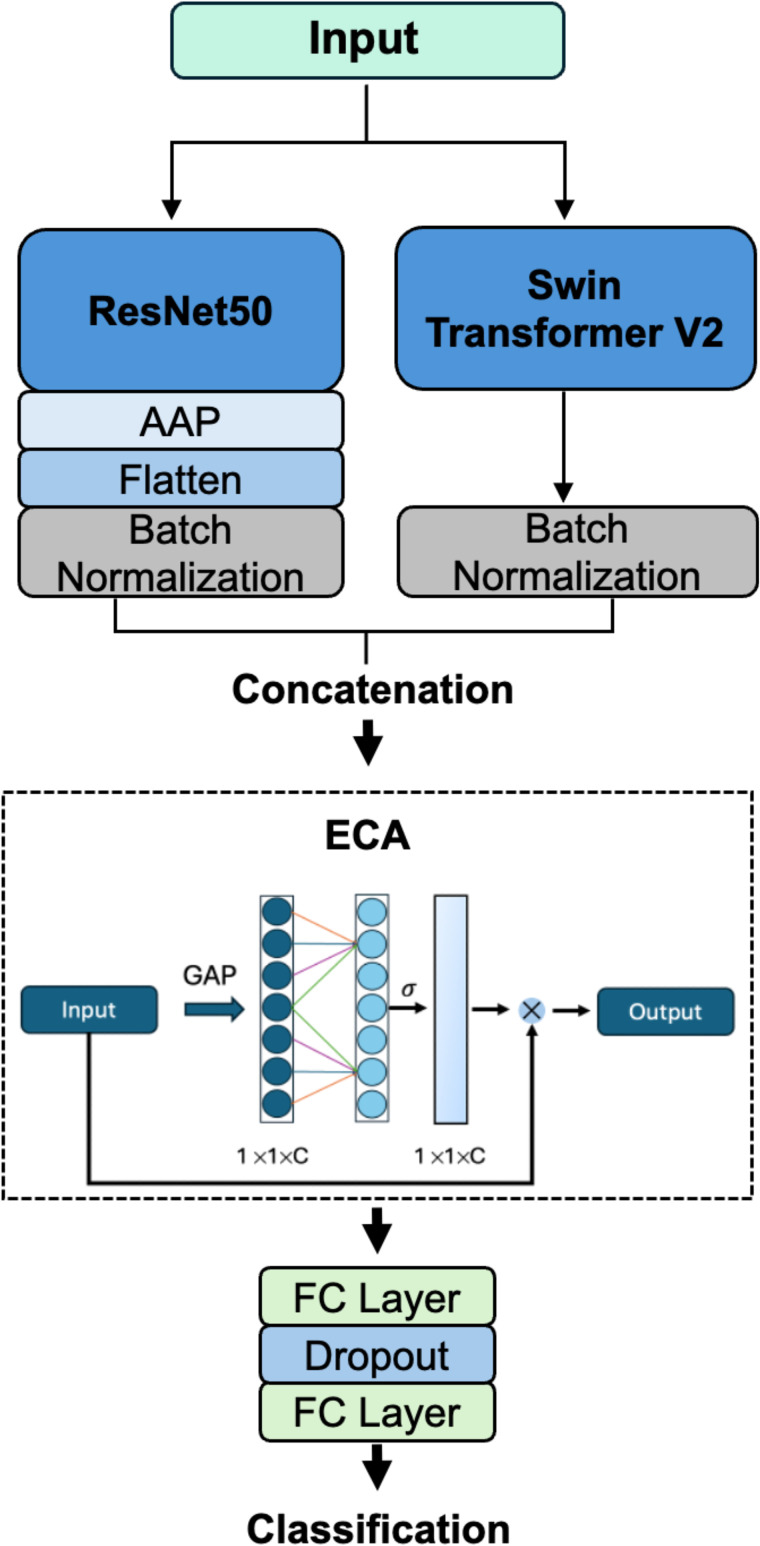
Visualization of the proposed architecture. The two architectures, ResNet50 and Swin Transformer V2, each extract key features from the input images. The respective one-dimensional vectors are then fused. The fused vector is then processed by the Efficient Channel Attention (ECA) module [39], which highlights the most relevant features. This enhanced vector is passed through two fully connected layers to produce the final classification output.

The hybrid architecture integrates convolutional and transformer-based components, leveraging ResNet50 to extract fine-grained local features and Swin Transformer V2 to model broader spatial context. This combination is particularly advantageous for aerial railway imagery, where detailed object structures such as obstacles or track elements must be interpreted within their larger environmental context. The network is initialized with pretrained ImageNet weights, which promote faster convergence and improved generalization [29]. ResNet50 has been shown to be a robust and scalable backbone [30], with a structural design that helps reduce overfitting [31], thereby enhancing its generalization capabilities [32]. The Swin Transformer V2 enables our hybrid architecture to analyze the relationship between local and global image components [33]. This capability is crucial in the specific scenario as it enables the precise classification of an intrusion by small objects that are difficult to distinguish from other rail components, particularly the rail bed. Additionally, Swin Transformer is both efficient and easy to scale, as its computational power increases linearly in accordance with the resolution of the image [34]. The structure of the network is shown in Fig 2. ResNet50 generates deep feature maps from the input image. The output of Swin Transformer V2 is a one-dimensional vector. This feature map (**F**_ResNet50_) is first reduced using adaptive average pooling and then transformed into a one-dimensional vector through flattening. Equation 1 illustrates this process.


$$\begin{array}{c} {𝐟}_{\text{ResNet50}}=\text{Flatten}(\text{AAP}({𝐅}_{\text{ResNet50}})) \end{array}$$ (1)

Afterward, both one-dimensional vectors are individually passed through batch normalization to stabilize and normalize the intermediate representations [35]. To fuse the batch-normalized vectors (${\hat{f}}_{\text{ResNet50}}$ and ${\hat{f}}_{\text{Swin}}$) the concatenation function is used. Since the spatial dimensions have been removed, fusion is performed only along the channel axis *C*. The equation for the one-dimensional concatenation function is given [36]:


$$\begin{array}{c} \text{Concatenate}({\hat{f}}_{\text{ResNet50}},{\hat{𝐟}}_{\text{Swin}})=𝐎\in {\mathbb{R}}^{C_{1}+C_{2}} \end{array}$$ (2)

Subsequently, the ECA attention module is applied to emphasize the most relevant regions of the fused features [37]. ECA offers an effective attention mechanism at relatively low computational cost. The resulting enhanced feature vector produced by ECA serves as input to two fully connected layers, with a dropout layer placed in between to reduce overfitting. This design ensures that the classifier benefits from the diverse representational strengths of the fused features, enhancing its robustness in complex image classification tasks.

#### 3.1.2 Swin transformer V2.

CNNs are specialized in capturing and extracting local features in images. In some cases, local feature extraction disrupts the relationship of the local feature to the global image composition. The Swin Transformer does not have these limitations. This architecture concentrates on the global and local relationship of features [33]. The Swin Transformer starts by splitting the input image into non-overlapping patches, treating each patch as a single token and projecting its RGB values via a linear layer to a higher-dimensional space [34]. By merging neighboring patches together, the architecture builds a hierarchical, structured feature map. That increases channel dimensions at each stage [34].

**Fig 3:**
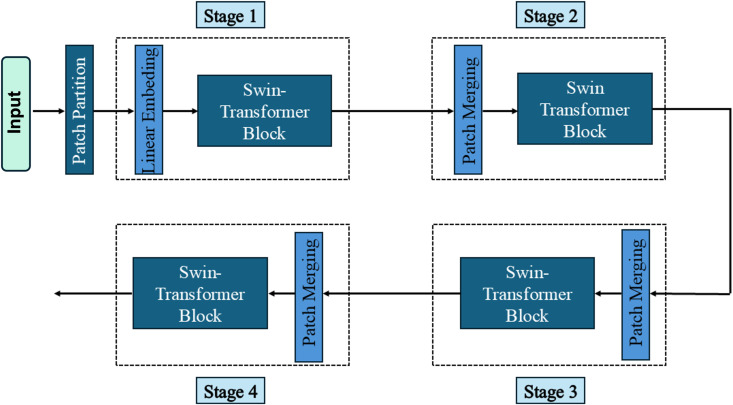
Simplified visualization of the general structure of a Swin Transformer [34]. The architecture consists of four stages, each containing a Swin Transformer block, which is designed to efficiently extract image-based features by combining local and global contextual information.

**Fig 4:**
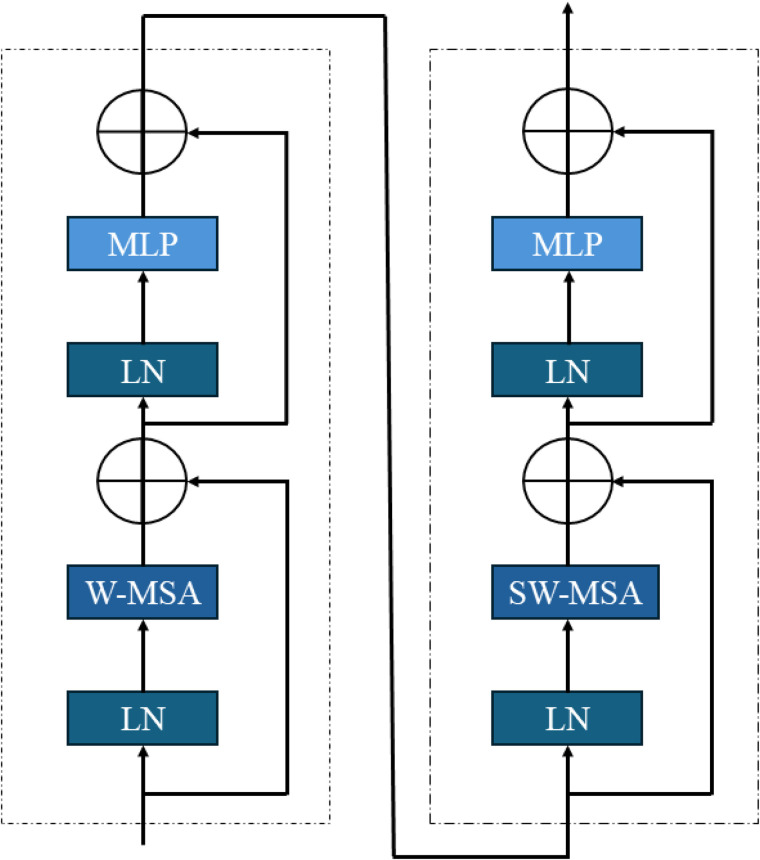
Structure of two consecutive Swin Transformer blocks [34]. The first block applies window-based multi-head self-attention (W-MSA), while the second uses shifted window attention (SW-MSA). Both blocks include layer normalization (LN), a multi-layer perceptron (MLP), and residual connections for stable and efficient feature extraction.

Swin Transformer V2 offers an approach that requires relatively low computing power compared to similar self-attention methods to recognize global and local relationships. To process cross-window information, the partitioning of windows is shifted between consecutive layers, so that windows in one layer overlap with those in the previous layers [34]. The network, as shown in Fig 3, consists of four stages, each stage contains its own Swin Transformer block. Each block, as shown in Fig 4 includes layer normalization (LN), followed by window-based or shifted window-based multi-head self-attention (W-MSA & SW-MSA) and a multilayer perceptron (MLP) with GELU activation. Residual connections are included throughout [34]. Between the stages, each block merges the patches and further downsamples the feature map and increases the feature dimensions. The computing power this architecture requires depends on the image size because the power the self-attention modules require linearly grows with the image size. This renders the architecture very efficient and scalable to high-resolution images [34]. The Swin Transformer’s global modeling capabilities are particularly beneficial for classifying objects on rails, where objects often need to be interpreted within a broader spatial context. The model improves the classification of obstacles and track-related elements by capturing global relationships, which allows it to handle objects of varying scales and complex surroundings. This makes it ideal for robust object recognition.

#### 3.1.3 ResNet50.

**Fig 5:**
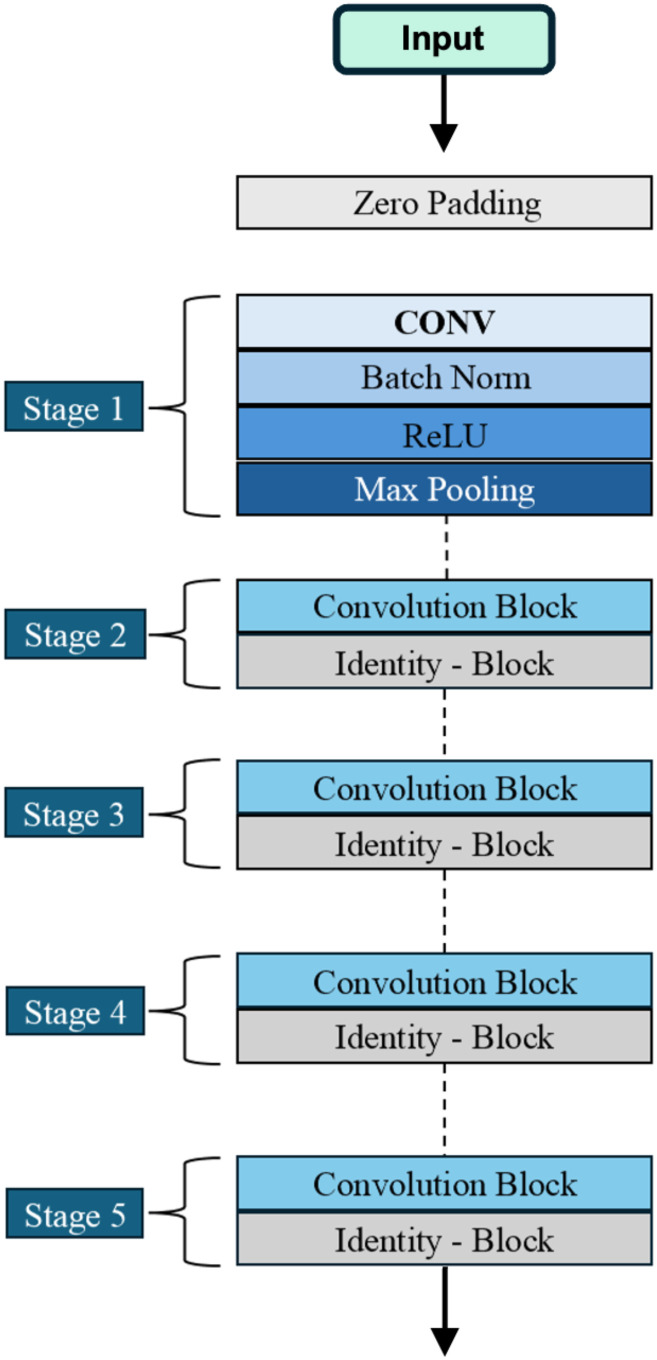
Visualization of the ResNet50 architecture, which forms part of the hybrid model. With its deep residual blocks and 3×3 convolutional layers, ResNet50 enables robust extraction of local features, making it ideal for the precise recognition of object-specific details in the track bed area.

ResNet50 is an architecture particularly well suited for this problem domain due to its robust feature extraction capabilities and proven stability. Its design, shown in Fig 5, makes it especially effective as a local feature extractor. Introduced by He et al. [31], ResNet50 is a powerful deep CNN designed for image classification. It is a residual network architecture that consists of 50 layers, organized into four main components: convolutional layers, identity blocks, convolutional blocks, and fully connected layers [38]. A key feature of ResNet50 is its identity mapping capability. Identity blocks enable certain layers to be bypassed if they are unnecessary, which helps mitigate overfitting [31]. The convolutional layers are responsible for extracting features from input images, while the identity and convolutional blocks process and transform these features. In stages two through five, each contains one convolutional block composed of 1×1 convolutions for dimensionality reduction and expansion, and a 3×3 convolution for feature extraction. Each convolutional layer is followed by a batch normalization layer. The number of identity blocks varies across stages: stage 2 has two blocks, stage 3 has four blocks, stage 4 has six blocks, and stage 5 has three blocks. During training, ResNet prioritizes learning low-level features before progressing to more complex representations, which improves training efficiency, stability, and generalization performance [32]. For this study, the top layer of the base model is omitted.

#### 3.1.4 Efficient channel attention.

**Fig 6:**
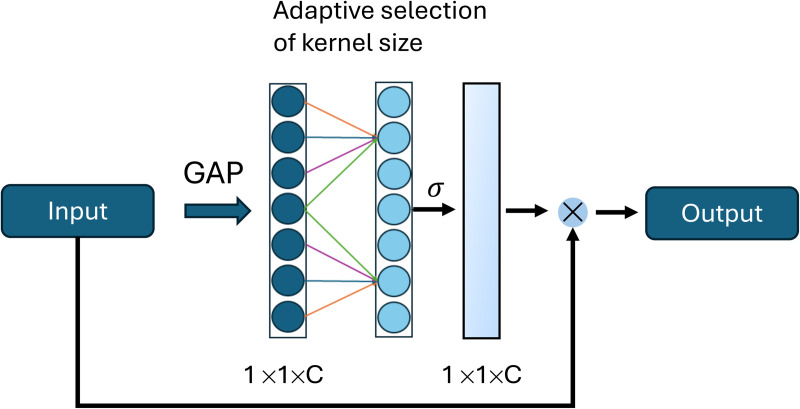
Schematic illustration of the architecture of Efficient Channel Attention [39].

Recent advances in deep learning for railway obstacle detection have seen an increasing incorporation of attention mechanisms to improve accuracy, particularly in challenging scenarios involving small, distant, or low-contrast objects [37]. In comparison to the earlier developed SE-Block attention module, the Efficient Channel Attention (ECA) module (Fig 6) offers some improvements. First, ECA does not use complex non-linear calculations; instead, it utilizes a 1D Convolution without the use of dimensional reduction [39]. The basic function of ECA starts with Global Average Pooling (GAP) without dimensional reduction, which captures local-cross channel interactions by choosing every channel with each k neighbors [39].

This method ensures the effectiveness and efficiency of the model. SE-Block requires high computational power because the calculations are mainly done by a fully connected layer (FC), ECA does not require comparable high computational power due to the calculation of an adaptive kernel size through a 1D convolutional layer. The adaptive kernel size is calculated with the equation below [39]:


$$\begin{array}{c} k=\psi (C)={\left|\frac{\log_{2}(C)}{\gamma }+\frac{b}{\gamma }\right|}_{\text{odd}} \end{array}$$ (3)

The number of channels is represented by the value of *C*, while γ represents the scale factor and *b* the offset. Finally, the result is rounded to the nearest odd number [39]. To highlight important areas on the original input, the result after the 1D convolutional layer is multiplied with the original input values [39]. ECA is a lightweight module capable of emphasizing the most relevant features. This is particularly important after the fusion of feature maps, where global information from the Swin Transformer V2 and more local features from ResNet50 are combined. ECA helps automatically identify and enhance the most informative channels within this mixed representation.

### 3.2 Process of training

**Fig 7:**
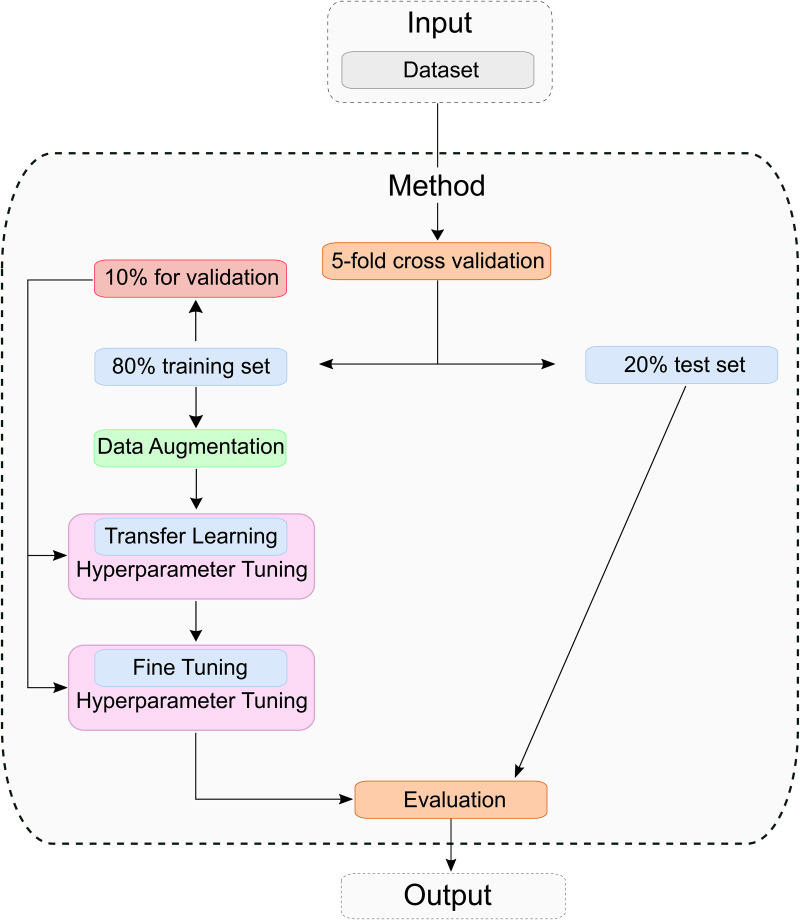
Training and evaluation approach: The dataset is split into a training set and a test set. Data augmentation is applied to the training set, followed by model training using transfer learning and fine-tuning. Finally, the resulting model is evaluated on the test set.

The entire training process is shown in Fig 7. Before model training, a stratified 5-fold cross-validation was applied using the scikit-learn library [40] to divide the dataset into five equally sized subsets. This method was chosen to ensure that each fold maintains the original class distribution, unlike standard k-fold cross-validation, which may produce biased results due to class imbalance. In each iteration, four folds (80%) are used for training, and one fold (20%) is held out for testing. This process is repeated five times, rotating the test fold each time to ensure robust performance evaluation. Within the training set of each fold, 10% is further reserved for validation and hyperparameter tuning. All images are resized to 256×256 pixels with three RGB channels and normalized using the ImageNet mean and standard deviation. To reduce overfitting and improve generalization, the training data is augmented using RandomRotation(±10°), RandomResizedCrop(scale=(0.9, 1.0)), RandomAffine(degrees = 0, translate=(0.1, 0.1)). These transformations account for variability in image scale and position, making the model more robust to input variations. Each model is trained for a maximum of 100 epochs. The training consists of two phases: transfer learning and fine-tuning. During transfer learning, the pretrained feature extraction layers (based on ImageNet weights [29]) are frozen, while the fully connected layers remain trainable. In the fine-tuning phase, the entire model is updated. Hyperparameter optimization is performed separately for both training phases using Optuna with a Tree-Structured Parzen Estimator (TPE) approach. For each fold, 20 trials are conducted, and the configuration that yields the lowest validation loss is selected. Each trial is allowed to run up to 100 epochs. To avoid overfitting and reduce computational overhead, early stopping is implemented, terminating training if the validation loss does not decrease over ten consecutive epochs.

After identifying the optimal hyperparameters, a final transfer learning model is trained using the same configuration as during optimization. The model with the lowest validation loss is saved and used as the base for the subsequent fine-tuning phase. In fine-tuning, the hyperparameters (see Table 1) are re-optimized, and additional feature extraction layers are unfrozen to enable further domain adaptation. Again, the best-performing model based on validation loss is stored.

**Table 1:** Overview of the hyperparameters used for hyperparameter tuning. TL = Transfer learning; FT = Fine-Tuning‌‌.

Hyperparameter	Minimum Value	Maximum Value	Step	TL or FT
Dropout	0.1	0.5	0.05	TL, FT
Learning Rate Transfer Learning	10^−4^	10^−2^	calculated logarithmically	TL
Learning Rate Fine-Tuning	10^−6^	10^−4^	calculated logarithmically	FT
Weight decay	10^−5^	10^−3^	calculated logarithmically	TL, FT
1. FC layer units	128	1024	128	TL, FT
Batch size	–	–	8, 16 or 32	FT
Optimizer	–	–	AdamW, SGD	TL

### 3.3 Evaluation metrics

After training, the final model is evaluated on the 20% test set using the following performance indicators such as accuracy, balanced accuracy, true positive rate (TPR or recall), true negative rate (specificity), positive predictive value (precision), Negative predictive value, Cohen’s Kappa, and F1-Score. The following metrics refer to the multiclass case, taking into account the class weighting, *n*_*i*_ represents the number of samples per class, and *S* denotes the total number of samples. Accuracy is the most commonly used performance metric that describes the overall effectiveness of the classifier. It is calculated by the set of all the instances: the truly positive instances, the truly negative instances and the falsely positive instances [41].


$$\begin{array}{c} \text{Accuracy}=\frac{\text{TP}+\text{TN}}{\text{TP}+\text{TN}+\text{FP}+\text{FN}} \end{array}$$ (4)

Another commonly used performance metric is the balanced accuracy. This is used especially in cases where highly imbalanced classes are given. For multiclass problems like the one this study outlined, it is calculated as the average value of all TPRs [44]. *N* denotes the number of classes.


$$\begin{array}{c} \text{Balanced Accuracy}=\frac{1}{N}\sum_{i=1}^{N}\frac{TP_{i}}{TP_{i}+FN_{i}} \end{array}$$ (5)

An important measurement is the TPR, sometimes called recall. It is the performance metric that calculates the overall efficiency of the classification. The TPR indicates what proportion of the actual positive cases were correctly recognized as positive by the model [41,42]. The true negative rate (TNR) is the counterpart to the TPR. It indicates what proportion of the actual negative cases were correctly recognized by the model. TPR is calculated by [43,44]:


$$\begin{array}{c} \text{TPR}=\sum_{i=1}^{N}\frac{n_{i}}{S}\cdot \frac{TP_{i}}{TP_{i}+FN_{i}} \end{array}$$ (6)

$$\begin{array}{c} \text{TNR}=\sum_{i=1}^{N}\frac{n_{i}}{S}\cdot \frac{TN_{i}}{TN_{i}+FP_{i}} \end{array}$$ (7)

The positive prediction value (PPV) or referred to as precision, describes the agreement of data class labels with those identified by the classifier [45]. It provides a measurement for the prediction accuracy for the positive class [41]. The counterpart is the negative prediction value (NPV) refers to the proportion of true negative predictions among all predicted negatives [46].


$$\begin{array}{c} \text{PPV}=\sum_{i=1}^{N}\frac{n_{i}}{S}\cdot \frac{TP_{i}}{TP_{i}+FP_{i}} \end{array}$$ (8)

$$\begin{array}{c} \text{NPV}=\sum_{i=1}^{N}\frac{n_{i}}{S}\cdot \frac{TN_{i}}{TN_{i}+FN_{i}} \end{array}$$ (9)

A popular performance metric in cases with a class imbalance, the F1-Score is used to evaluate the model’s performance in regard to the relations between data’s positive labels and those recognized by the classifying model [45]. It balances precision and recall by calculating a harmonic mean [41]. The formula used for the multiclass case is provided by [47]:


$$\begin{array}{c} \text{F1-Score}=\sum_{i=1}^{N}\frac{n_{i}}{S}\;2\cdot \frac{PPV_{i}\cdot TPR_{i}}{PPV_{i}+TPR_{i}} \end{array}$$ (10)

Cohen’s Kappa (κ) measures the level of agreement between predicted and true labels while accounting for the agreement that could occur by chance [48]. In this metric, *P*_*o*_ denotes the observed agreement, and *P*_*e*_ represents the agreement expected by random chance.


$$\begin{array}{c} \kappa =\frac{P_{o}-P_{e}}{1-P_{e}} \end{array}$$ (11)

### 3.4 Dataset

This study utilizes the UAV-RSOD dataset [12], which was specially designed for railway obstacle detection. The railroad where the images were taken has a length of 378m and is located at the junction railway station in Tiruchirapalli, Tamil Nadu, India. The different railway segments were captured by a “DJI Phantom 4 PRO UAV” equipped with a “Sony DSC-RXC1RM2, 20MP” full resolution camera. To achieve better results, the UAV was modified with an additional camera set to take the images. During the flight, the UAV utilizes a camera setting of 8.8 mm focal length and 1920×1080 HD video recording at 50 Mbps. The dataset consists of 2,003 images divided into six classes: Iron rod, Boulder, Person, Branch, Jerrycans, and Barrel. The following Table 2 shows the distribution of the 2,003 images into the different classes.

**Table 2:** Overview of the six classes with descriptions and number of images per class used in this study.

Class	Description	Number of Images
Class 1	Iron Rod	346
Class 2	Boulder	208
Class 3	Branch	548
Class 4	Jerrycan	395
Class 5	Person	234
Class 6	Barrel	272
**In total**		**2,003**

### 3.5 Setup

For training and testing the architecture, an NVIDIA L40S GPU with 48 GB of memory with PyTorch 2.5.0 is used. Furthermore, Python version 3.11.7 and CUDA version 12.4.1 were used. The hybrid architecture was trained for a maximum of 100 epochs using either AdamW or SGD, depending on which optimizer achieved the best performance during hyperparameter tuning. Training was performed in phases, including transfer learning and fine-tuning. To identify the optimal parameters for transfer learning and fine-tuning, hyperparameter tuning was performed in 20 trials using Optuna (version 4.2.1). To avoid overfitting and save computation time, the callback function *early stopping* with a *delta* = 0.001 was used, which stops the training after 10 consecutive epochs in which the validation loss has not decreased. Scikit-learn (version 1.5.2) was utilized for stratified cross-validation and the computation of performance indicators. Throughout the entire training and validation process, the images were converted to a resolution of 256×256 pixels.

## 4 Results

**Fig 8:**
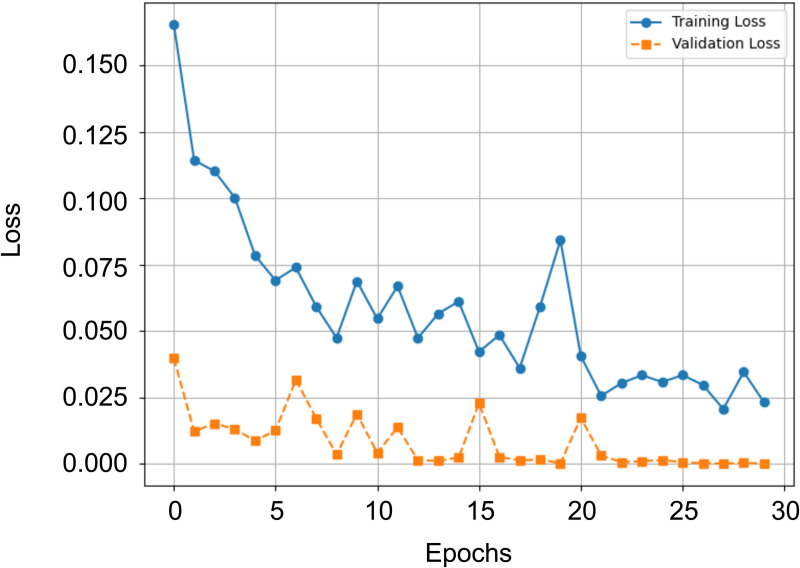
Illustration of a training and validation loss curve during the training process. The process was terminated by early stopping.

In this section, we present the results achieved by the proposed hybrid architecture. Table 3 summarizes the classification performance across all individual folds as well as the overall average. The model was evaluated using a stratified 5-fold cross-validation setup. Fig 8 shows the training‌‌ loss and validation loss curves for one run of the cross-validation. The results clearly show that the model achieves a balanced accuracy ranging from 99.25% to 99.75%, with an overall average of 99.46%, setting a new benchmark for this classification task. Notably, Fold 2 consistently yields the highest performance across nearly all evaluated metrics, suggesting particularly strong generalization in that split. Balanced Accuracy is selected as the primary evaluation metric due to the class imbalance in the multiclass dataset. In imbalanced settings, standard accuracy may be dominated by majority classes and thus provide an overly optimistic performance estimate. Balanced Accuracy prevents performance on infrequent but safety-relevant classes from being masked by dominant categories, which is particularly important in railway obstacle detection.

**Table 3:** Results of the evaluation metrics of the proposed hybrid architecture‌‌.

Fold Metric	1	2	3	4	5	AVG
**Accuracy**	0.9925	**0.9975**	0.9925	0.9950	0.9950	0.9945
**Balanced Accuracy**	0.9939	**0.9985**	0.9915	0.9954	0.9938	0.9946
**TPR**	0.9939	**0.9975**	0.9915	0.9950	0.9950	0.9945
**TNR**	0.9982	**0.9996**	0.9978	0.9991	0.9991	0.9988
**PPV**	0.9927	**0.9976**	0.9927	0.9951	0.9951	0.9945
**NPV**	0.9982	0.9991	0.9987	0.9987	**0.9992**	0.9987
**Kappa**	0.9908	**0.9969**	0.9908	0.9939	0.9938	0.9933
**F1-Score**	0.9925	**0.9975**	0.9925	0.9950	0.9950	0.9945

On average, the TPR, PPV, and F1-score each reach 99.45%, indicating a high level of sensitivity and precision across classes. Even higher values are observed for the TNR and NPV, with averages of 99.88% and 99.87%, respectively. These results imply that the model is highly reliable in rejecting instances that do not belong to a given class, with minimal confusion between different categories. Additionally, the model achieves a Cohen’s Kappa score of 0.9933, reflecting an almost perfect level of agreement beyond chance. Furthermore, Table 5 provides an overview of the evaluation results for the proposed hybrid model, alongside the individual baseline architectures ResNet50 and Swin Transformer V2, as well as the hybrid variant without the ECA module. The results clearly show that the proposed hybrid model with ECA consistently achieves the highest values across all evaluated metrics, thereby emphasizing its superior performance and effectiveness compared to the other approaches.

**Table 4:** Optimal hyperparameters identified for transfer learning and fine-tuning across all five folds. Listed are learning rate, weight decay, dropout rate, dense layer units, batch size and optimizer.

Fold	I	II	III	IV	V
**Transfer Learning**					
Learning Rate	4.17e-04	1.32e-03	1.27e-03	5.64e-03	4.57e-04
Weight Decay	6e-06	2.91e-03	7.60e-05	3.65e-06	6.78e-05
Dropout Rate	0.20	0.20	0.25	0.25	0.10
Dense Layer	640	384	896	256	1024
Optimizer	AdamW	AdamW	AdamW	AdamW	AdamW
**Fine-Tuning**					
Learning Rate	1.37e-05	1.84e-05	6.21e-05	9.25e-06	2.53e-05
Weight Decay	3.0e-03	2.82e-04	4.1e-05	6.04e-05	4.49e-04
Dropout Rate	0.2	0.40	0.30	0.30	0.25
Batch Size	8	16	32	8	8
Optimizer	AdamW	AdamW	AdamW	AdamW	AdamW

**Table 5:** Comparison of the performance of the proposed hybrid architecture with and without ECA, alongside the baseline architectures ResNet50 and Swin Transformer V2.

Metric	ResNet50	Swin Transformer V2	Hybrid Architecture without ECA	Hybrid Architecture with ECA
**Accuracy**	0.9890	0.9880	0.9925	**0.9945**
**Balanced Accuracy**	0.9886	0.9889	0.9926	**0.9946**
**TPR**	0.9890	0.9880	0.9925	**0.9945**
**TNR**	0.9978	0.9973	0.9984	**0.9988**
**PPV**	0.9894	0.9882	0.9926	**0.9945**
**NPV**	0.9975	0.9970	0.9982	**0.9987**
**Kappa**	0.9865	0.9853	0.9908	**0.9933**
**F1-Score**	0.9890	0.9880	0.9925	**0.9945**

When considering the balanced accuracy, ResNet50 yields the lowest performance, followed by Swin Transformer V2. The hybrid architecture without ECA still outperforms both baseline models across all metrics. The results further demonstrate the effectiveness of the ECA attention module. Table 6 compares the results of the hybrid architecture with other baseline models DenseNet121, EfficientNetB4, and MobileNetV2, where it is also clearly shown that the hybrid architecture consistently achieves the highest values for all metrics.

**Table 6:** Comparison of the performance of the proposed hybrid architecture with the baseline models DenseNet121, EfficientNetB4 and MobileNetV2.

Metric	DenseNet121	EfficientNetB4	MobileNetV2	Hybrid Architecture with ECA
**Accuracy**	0.9875	0.9835	0.9865	**0.9945**
**Balanced Accuracy**	0.9878	0.9853	0.9860	**0.9946**
**TPR**	0.9875	0.9835	0.9865	**0.9945**
**TNR**	0.9972	0.9967	0.9971	**0.9988**
**PPV**	0.9879	0.9840	0.9867	**0.9945**
**NPV**	0.9969	0.9956	0.9971	**0.9987**
**Kappa**	0.9847	0.9798	0.9834	**0.9933**
**F1-Score**	0.9875	0.9835	0.9865	**0.9945**

**Fig 9:**
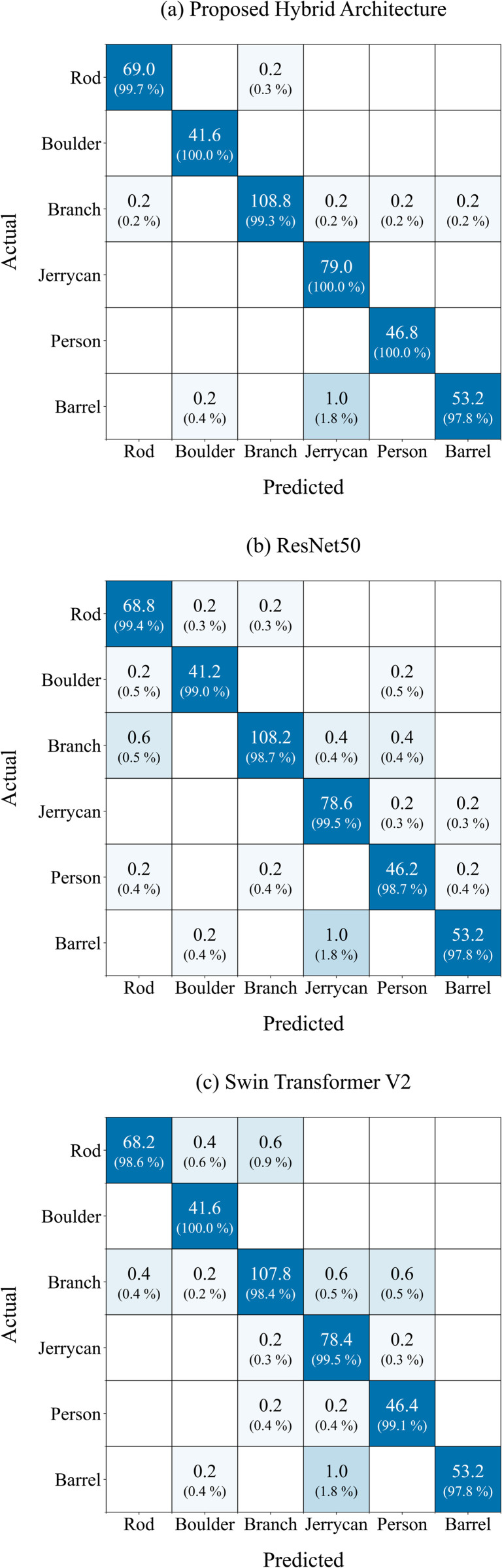
The average confusion matrices across all five folds of the proposed hybrid architecture, ResNet50, and Swin Transformer V2.. Absolute values, along with their corresponding percentages, are shown..

In addition to the performance metrics, Fig 9 (a) presents the average confusion matrix of the proposed hybrid architecture. This figure shows the absolute classification values aggregated across all five folds, rounded to two decimal places. Below each absolute value, the corresponding row-normalized percentage is displayed in parentheses. The class Iron Rod was correctly classified in 99.7% of the cases, with only 0.3% of its instances being misclassified as Branch. The classes Boulder, Jerrycan, and Person were classified with 100% accuracy, showing no misclassifications across any fold. Most of the misclassifications occurred in the class Branch, which was occasionally confused with Iron Rod, Jerrycan, Person, and Barrel, resulting in comparatively lower percentages across those categories. The lowest classification accuracy was observed for the class Barrel, with 97.8% correctly identified. The most common misclassification for Barrel was as Jerrycan (1.8%), followed by Boulder (0.4%). (b) and (c) of Fig 9 show the basic models ResNet50 and Swin Transformer V2. It is striking that the class Barrel is classified correctly with the same probability for each architecture. To ensure reproducibility, Table 4 lists the optimal hyperparameters used for the proposed hybrid architecture. During the transfer learning phase, the learning rate ranged from 4.57e-04 to 5.64e-03, and the weight decay values were between 3.65e-06 and 2.91e-03. The dropout rate ranged from 0.10 to 0.25 during transfer learning and from 0.20 to 0.40 in fine-tuning. The first dense layer contained between 256 and 1024 neurons. AdamW was consistently used as the optimizer. Learning rates during fine-tuning varied between 9.25e-06 and 6.21e-05, with weight decay ranging from 4.1e-05 to 3.0e-03. Batch sizes of 8, 16, and 32 were used across the folds.

## 5 Discussion

### 5.1 Comparison of the results

Table 3 presents the results obtained by the hybrid architecture for each individual fold. The results are stable across all folds, as evidenced by the low variation, such as the standard deviation of ±0.0023 in balanced accuracy. Due to the use of stratified five-fold cross-validation as the evaluation technique, these results reflect the overall robustness of the proposed hybrid architecture. When compared to the baseline models shown in Table 5, the proposed hybrid model consistently achieves the highest scores. The ResNet50 architecture, in particular, includes skip connections and a substantial number of 3×3 convolutional layers, making it highly suitable for local feature extraction. The residual connections help stabilize the learning of features, which is especially beneficial for capturing small objects. Depending on how the UAV images are captured, large-scale scenes with multiple objects, background structures, and contextual information may also appear, further emphasizing the need for architectures that can capture both local and global features effectively. In such cases, local features alone are not sufficient to reliably detect objects. Swin Transformer V2 is an architecture that incorporates both local and global feature representations. However, as shown in Table 5, its performance in terms of overall accuracy, true positive rate (TPR), and F1-score is slightly lower than that of ResNet50, although it achieves a higher balanced accuracy. This indicates that, for the type of images used in this study, local features play a more critical role. A hybrid approach that integrates the strengths of both ResNet50 and Swin Transformer V2 proves to be highly effective. The hybrid model without the ECA module achieves a balanced accuracy of 99.25%, surpassing both ResNet50 (98.90%) and Swin Transformer V2 (98.80%). This clearly demonstrates that the fusion of local and global feature extractors within a single architecture yields better results than using either model in isolation. Furthermore, the inclusion of an attention mechanism such as ECA leads to an additional performance boost, confirming its value as a lightweight yet impactful enhancement for feature refinement and classification accuracy. Furthermore, Table 6 lists the results achieved by other baseline models DenseNet121, EfficientNetB4, and MobileNetV2, which are not part of the hybrid architecture. This shows that all architectures achieve lower results in balanced accuracy and other metrics compared to the baseline models Resnet50 and Swin Transformer V2 (Table 5). The hybrid architecture achieves the highest values in all metrics. In terms of balanced accuracy, the hybrid architecture achieves increases of 0.006/0.6 percentage points (pp) (ResNet50), 0.0057/0.57 pp (Swin Transformer V2), 0.0068/0.68 pp (DenseNet121), 0.0093/0.93 pp (EfficientNetB4), and 0.0086/0.86 pp (MobileNetV2). In summary, the proposed hybrid architecture leverages the strengths of two baseline models that achieve high scores compared to other baseline models. Furthermore, the hybrid architecture achieves the highest scores in the metrics compared to the baseline models.

A comparison of the results achieved by the hybrid architecture with relevant studies on this topic shows that previous literature in this area has focused primarily on the localization of obstacles in the track bed and less on classification. This includes methods such as segmentation [5,7,16,17] and object detection [6,9,18–25]. In contrast, the semantic interpretation and classification of obstacle types have received significantly less attention. Where classification is incorporated, it is usually limited to simple binary distinctions, rather than addressing the diverse set of obstacle categories encountered in real-world applications [26,28]. The present study challenges this perspective and demonstrates that achieving high classification accuracy across several relevant classes relies on the effective integration of both local and global feature representations. The average balanced accuracy achieved in this way represents a new, high-performance benchmark in a relevant yet currently underexplored area of research. The performance achieved by the proposed hybrid architecture with a balanced accuracy of 99.46%, which is specifically adapted to the characteristics of UAV imagery of the track bed, exceeds the previously reported mean precision of 96.88% by Chen et al. [26] and the accuracy of 85.2% by Kapoor et al. [28], even though a broader range of potential obstacle types was addressed for the first time. In addition, our results were validated using stratified five-fold cross-validation. This perspective, therefore, complements existing research.

### 5.2 Confusion matrix

Our proposed hybrid architecture achieved a very high balanced accuracy of 99.46%. Nevertheless, some object classes were still misclassified. The widest range of misclassifications occurred for the class Branch, which was incorrectly identified on average as Iron Rod, Jerrycan, Person, or Barrel, although each with a very low average of 0.2 misclassified images. Similarly, Iron Rod was misclassified as Branch with an average of 0.2 images, which, like the misclassifications of Branch, represents a very low error rate. The baseline architectures (Fig 9 (b) and (c)) show either a greater number of misclassifications in the mentioned classes, such as Iron Rod being confused with two classes instead of one, or higher misclassification rates for a specific class when compared to the proposed hybrid architecture. In general, the majority of the off-diagonal values are 0.00, indicating that the classes are well distinguishable. Moreover, the misclassifications within the classes are so minimal that no systematic errors or biases can be assumed. Overall, out of the six classes, three are recognized with 100% accuracy. The lowest recognition rate is observed for the class Barrel at 97.8%. A similar result is seen in the baseline architectures, along with a comparable pattern of misclassifications. The most frequent confusion occurs with Jerrycan, with an average of one misclassified image per fold, which is 0.8 higher than the second-highest misclassification rate. One possible explanation for this may be the visual similarity between the two objects. Both share a cylindrical shape, which sets them apart from elongated objects like iron rods, branches, or persons, as well as from circular forms such as boulders. Finally, when considering the unequal number of images per class, it becomes evident that despite the class imbalance, categories with fewer images (such as Boulder or Person) show no significant differences in accuracy or misclassification rates. The hybrid architecture is therefore able to achieve very good recognition performance even on imbalanced classes, which is an important capability for practical applications when rare classes occur that are present with fewer images.

### 5.3 Inclusion of a clear track class and real-world bias analysis

**Fig 10:**
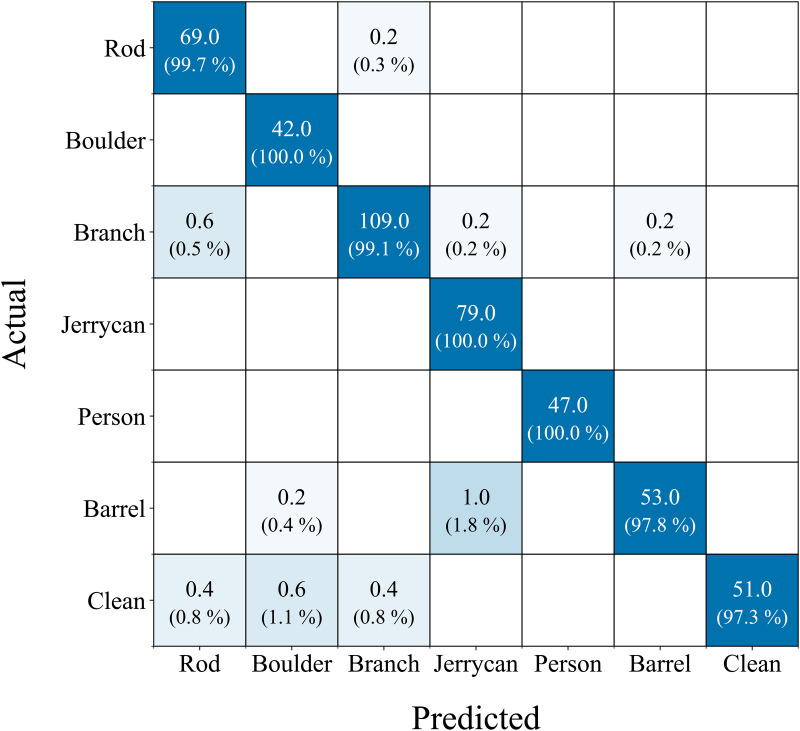
The average confusion matrix across all five folds of the proposed hybrid architecture, evaluated with an additional clean class. Absolute values along with their corresponding percentages are shown.

To mitigate the model bias toward “obstacle presence” for real use case, we have included a “Clear track” class as a separate analysis to demonstrate that our model does not exhibit frequent false alarms. The setup analyzed in the main study comprised only six classes. In reality, however, this case is not necessarily representative, as a large proportion of the images will show clean rail beds. To simulate this, 260 clean samples were generated from the dataset by selecting image sections from existing images that did not contain any objects. Fig 10 shows the results of the evaluation of our proposed hybrid architecture for this extended case.

The false negative rate compared to clean samples is particularly important for such safety-critical application scenarios, as false negatives would mean missed obstacles and thus potential operational risks. Our hybrid architecture shows not a single missed alarm across the entire cross-validation, which emphasizes its suitability for real-world applications. The results further show that the hybrid architecture achieves a very high balanced accuracy of 99.13% in this validation scenario. Three classes achieve perfect performance (boulder, jerrycan, and person). Thus, our architecture does not show a bias towards an obstacle presence.

**Fig 11:**
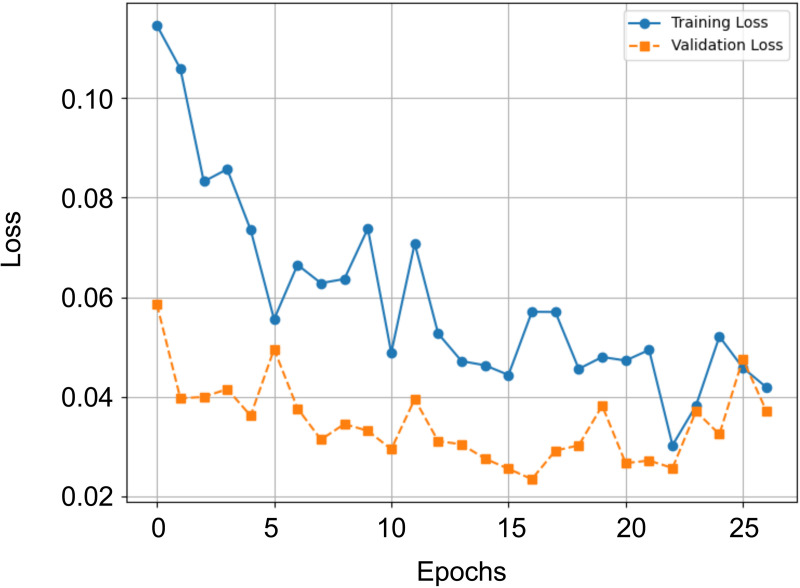
Training and validation loss curves of the proposed hybrid architecture across epochs with the new class “clean”. The process was terminated by early stopping.

To support our claims of model performance, Fig 11 shows the training and validation loss curve of our model under this evaluation setup.

After an initially high start, the training and validation loss curves show a steady decrease, indicating clean convergence. There is no significant deviation between the two curves, and the validation loss stabilizes at a low level. Early stopping correctly terminates training after both curves have stabilized, so there is no premature termination of training. The curves therefore support the key figures presented.

### 5.4 Practical implications

This study addresses a research problem that has received limited attention in the literature by tackling the high-performance classification of a wide range of obstacle types within the railway track bed. The model’s strong performance is the basis for its practical relevance. Deploying automated obstacle classification systems in railway environments requires the highest possible accuracy across all relevant object classes. An important question that remains to be addressed is where such systems should be installed. One option is to integrate them directly into trains. However, it must first be determined whether such an installation is technically feasible. Trains are often closed systems, and retrofitting them, if space is available, requires careful evaluation to ensure that the deep learning system does not interfere with existing onboard technologies. In addition, sufficient computational resources must be available to enable fast classification. Given the high speeds at which trains operate and their long braking distances, often several hundred meters [49], objects must be detected quickly to allow the train to come to a safe stop in time. Poor lighting conditions and curved tracks further complicate this, making the integration of deep learning systems more challenging. Another option is the installation of stationary systems that continuously monitor a specific area. These systems have the advantage of being managed in a decentralized manner. However, they are limited in that they can only cover a specific section of the track. Since railway networks can span several hundred kilometers, complete coverage with fixed installations would not be economically feasible. In addition, ongoing maintenance of these systems would be required. Therefore, this approach is more suitable for locations such as level crossings or areas near train stations. Another possibility is the use of drones, as illustrated in Fig 1. Railway sections can be inspected on demand, and the use of drones is more cost-effective than installing fixed systems. Several kilometers of track can be monitored with a single drone. However, if full coverage of an entire railway line is desired, multiple drones would be required, as both flight range and battery life are limited. Compared to fixed installations or integration directly into the train, this option is more advantageous due to its lower cost and the ability to perform predictive classification with timely alerts sent to the train. Furthermore, unlike fixed systems, drones can inspect objects more closely and from multiple angles, enabling more accurate and reliable classification.

## 6 Conclusion

Railways remain one of the most widely used and essential modes of transportation globally [50]. However, accidents are often caused by objects or individuals present on the tracks, making the safety of passengers and personnel a critical concern. While current research and industrial solutions mainly focus on detecting the presence or position of obstacles in the track bed, they often fall short in comprehensively classifying the full range of relevant obstacle types. This study addresses that gap by leveraging the unique visual characteristics found in track bed imagery through the application of a hybrid deep learning architecture. By integrating both convolutional and transformer-based components and employing the Efficient Channel Attention module, the architecture is specifically designed to capture and enhance both local and global spatial relationships within the images, enabling more precise and context-aware classification. The proposed approach was evaluated on a dataset containing 2,003 images distributed across six object classes, using stratified 5-fold cross-validation to ensure robust and balanced evaluation. Achieving an average balanced accuracy of 99.46%, our hybrid model sets a new performance benchmark in this problem domain, demonstrating its effectiveness in handling the complexity and variability inherent in real-world track bed conditions. Through evaluation, we show that combining the strengths of multiple architectural characteristics leads to significantly improved results compared to using standalone architectures. Overall, our study demonstrates the strong potential of modern hybrid architectures in accurately and reliably classifying diverse types of track bed obstacles. This advancement represents a meaningful step toward fully automated railway monitoring, contributing to increased safety, reduced operational risk, and more efficient transportation systems.

### 6.1 Limitations

In this study, we presented a high-performing and novel hybrid deep learning architecture. While the achieved results demonstrate the effectiveness and robustness of the proposed method, it is important to acknowledge certain limitations of this work. The dataset used in this study contains six predefined object classes, and therefore, the strong performance of the hybrid architecture is currently restricted to this limited set of objects. In real-world scenarios, however, additional and potentially unexpected objects may appear on railway tracks, whose detection accuracy is not evaluated within the scope of this dataset. As a result, the generalizability of the model to a broader range of obstacles remains uncertain. Moreover, the images in the dataset were captured from a top-down aerial perspective, which provides two-dimensional visual information. This limits the model’s ability to capture depth cues or three-dimensional spatial relationships. In addition, the images are confined to specific sections of the railroad and do not cover more complex track configurations, such as level crossings or areas with multiple parallel rails.

### 6.2 Future work

Building on the outlined limitations, several promising directions for future research emerge. To enhance the external validity and generalizability of the proposed approach, future studies could aim to evaluate the model across additional domains that use UAV-based datasets, such as applications in traffic monitoring or infrastructure inspection. Moreover, future research could address the limited variability in environmental conditions, such as weather and time of day, by leveraging generative models like Generative Adversarial Networks or diffusion models. These models can be used to synthetically augment datasets with realistic variations, helping to mitigate the lack of diversity in the training data. In addition, generative methods can support the resolution of dataset imbalances by generating more representative samples for underrepresented object classes or scenarios. In addition, the current architecture for processing video data could be extended by adapting it to the processing of temporal information, as in [51]. This would enable the model to be applied not only to static images but also to dynamic visual input, making it suitable for video-based monitoring.
